# Efficacy and Safety of 0.25% Timolol Gel in Healing Split-Thickness Skin Graft Site

**DOI:** 10.22037/ijpr.2020.114565.14915

**Published:** 2021

**Authors:** Amirhossein Ghanbarzamani, Ebrahim Salehifar, Abdolreza Jafarirad, Mohammad Hossein Hesamirostami, Ali Bagherzadehsaba, Majid Saeedi, Monireh Ghazaeian, Ghasemali Khorasani, Mahmood Moosazadeh

**Affiliations:** a *Department of Clinical Pharmacy, Faculty of Pharmacy, Mazandaran University of Medical Sciences, Mazandaran, Iran. *; b *Department of Surgery, Zare Psychiatry and Burn Hospital, Mazandaran University of Medical Sciences, Sari, Iran. *; c *Department of Pharmaceutics, Faculty of Pharmacy, Mazandaran University of Medical Sciences, Sari, Iran. *; d *Department of Plastic Surgery, Imam Khomeini Hospital, Tehran University of Medical Science, Tehran, Iran. *; e *Health Sciences Research Center, Addiction Institute, Mazandaran University of Medical Sciences, Sari, Iran.*

**Keywords:** Beta-blockers, Epithelization, Efficacy, Wounds, Burn

## Abstract

As a common intervention among burn patients, skin graft has some risks such as infections and delay of wound healing. The aim of this study was to assess the efficacy and safety of topical 0.25% Timolol Gel (TG) in promoting wound healing in split-thickness skin graft donor sites.

We conducted a double-blind, randomized clinical trial to assess re-epithelialization time, the level of pain based on the Visual Analog Scale (VAS), and the wound infection incidence. The scar status was also evaluated by the Vancouver Scar Scale (VSS) and the Patient and Observer Scar Assessment Scale (POSAS). Totally, 64 patients were randomly assigned to the study groups. The two groups showed a significant difference in healing time (14.5 ± 3.2 *vs*. 11.5 ± 2.3 days, *P < *0.001). No infection occurred in either group, and 3 cases of transplant rejection were observed in the placebo group. The VAS was significantly different on days 1, 2, 3, 4, and 7 (*P < *0.05). In the third month, the results showed a significant difference in terms of VSS (*P = *0.005). Topical TG, due to its favorable effects on wound healing and pain reduction, can be administered as a therapeutic agent in patients with a skin graft.

## Introduction

Skin grafting is performed by removing the skin from the donor site to cover the area where the skin is missing. The skin removed from the transplant donor site may contain epidermis or part of the dermis or both of them; the two cases are called split-thickness skin graft (STSG) and full-thickness skin graft (FTSG), respectively (1). STSGs are usually less aesthetically pleasing, and patients experience more pain due to donor sites than FTSG (2).

One of the most important issues in postoperative skin management is related to caring for the wound donor site since most patients have more discomfort on the donor site than on the recipient site (3).

STSG is a common method for patients with burns and those in need of plastic surgery. Wounds caused by skin grafts are at risk of infection or delayed wound healing, which should be well managed (4). 

After transplantation, a skin graft protects the wound from the environment, temperature, pathogens, and excessive water loss (5).

Timolol, a derivative of propanolamine, blocks the β-adrenergic receptor non-selectively.

*In-vivo* and *in-vitro* studies have shown that it accelerates wound epithelialization by blocking β2-Adrenergic receptors within the epidermis (6). Timolol has a low cost and is a non-invasive tool for healing wounds (7).

β2-Adrenergic receptors (B2AR) are present in various organ systems, one of which is the skin. These receptors are found on keratinocytes, fibroblasts, and melanocytes and may also play a role in the pathophysiology of dermatological diseases such as vitiligo, atopic eczema, and psoriasis (8, 9).

In acute partial-thickness donor site wounds, the presence of B2AR antagonists heals the wound because of the migration of keratinocytes (9-11).

One of the important processes for wound healing is epithelialization, in which the activation of β2-Adrenergic receptors delays wound healing by reducing the migration of keratinocytes via several mechanisms like activating serine/threonine phosphatase 2A. B2AR is involved in angiogenesis and proliferation of dermal fibroblasts. In contrast, β-blockers can accelerate epithelialization and healing by increasing keratinocytes’ migration (9, 10 and 12).

In β-blockers, propranolol is used in mice with chronic stress, and high levels of catecholamines cause a delay in wound healing, which could be prevented by administering high doses of propranolol (7, 13).

Healing skin wounds involves a complex process. It includes the migration of fibroblasts and keratinocytes and their proliferation by stimulating cytokines and growth factors. During this process, we see a wound healing pattern including inflammation, proliferation, maturation, and wound regeneration (6). Collagen acts as the main protein in the extracellular matrix, which comprises amino acids and plays a role in the integrity of the dermis and tissues (14, 15). Malnutrition is another factor that considerably affects wound healing (14, 15). One of the useful indicators for evaluating nutritional status is albumin level, and malnutrition can be diagnosed with low serum albumin level (16, 17). Malnutrition also causes anemia, which delays wound healing (18).

The aim of this study was to assess the efficacy and safety of topical 0.25% TG in promoting wound healing in STSG donor sites.

## Experimental

This double-blind, randomized clinical trial was conducted in Zare Hospital, a tertiary referral center affiliated with Mazandaran University of Medical Sciences (MAZUMS). Between February 2020 and May 2020, we recruited a total of 385 patients with burn and reconstructive plastic surgeries who met the eligibility criteria. Using a random-number–generating scheme based on permuted-block randomization, we divided the patients into two groups. The study was approved by the Ethics Committee of MAZUMS (IR.MAZUMS.REC.1398.5605) and registered in the IRCT database (IRCT20090613002027N18). The written informed consent was obtained from all patients or their guardians. The inclusion criteria were age over 18 years and is a candidate for STGS. On the other hand, the exclusion criteria were pregnancy, breastfeeding, use of systemic drugs (*e.g*., immune-suppressive drugs) which impede wound healing, use of a topical or systemic β-blocker drug other than the study drug, severe coagulation disorders such as deficiency of coagulation factors and immunological thrombocytopenic purpura (ITP), severe uncontrolled systemic comorbidities (such as diabetes, asthma, chronic obstructive pulmonary disease, and heart block), hypersensitivity to gel or Timolol, and patients’ unwillingness to cooperate.

**Figure 1:**
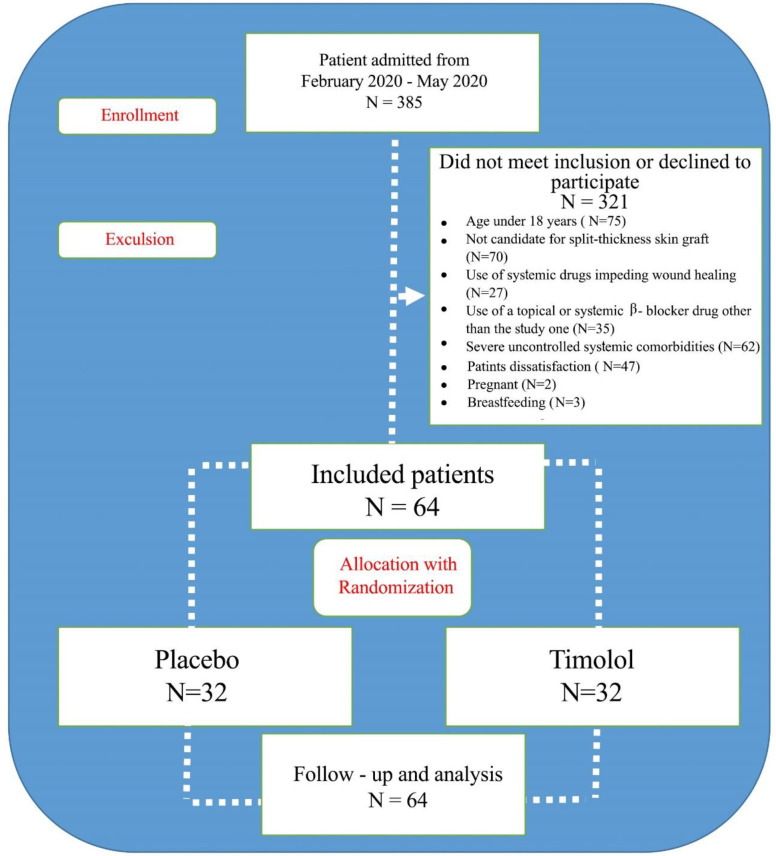
The study consort diagram

Of the 385 screened patients, 321 were excluded as they did not meet the eligibility criteria or declined to participate. The remaining 64 patients were randomized to receive TG or placebo, which was similar to TG except that it had no active ingredient (Figure 1).

**Figure 2:**
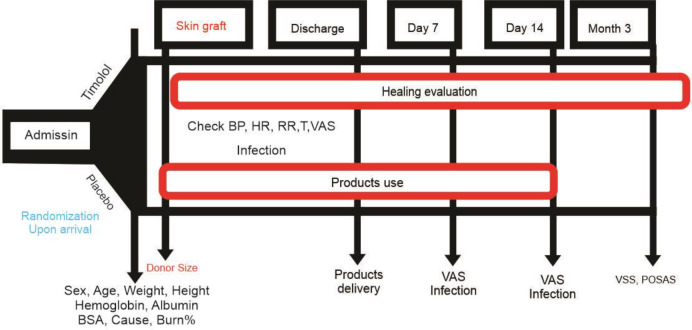
Patients monitoring plan. BP: Blood Pressure; HR: Heart Rate; RR: Respiratory Rate; T: Temperature; VAS: Visual Analogue Scale; BSA: Body Surface Area; VSS: Vancouver Scar Scale; POSAS: Patient and Observer Scar Assessment Scale

On hospital admission, the size and degree of the area needing skin repair for reconstructive plastic surgery were determined. Complete blood count and routine biochemical assays, including serum albumin and electrolytes, were analyzed. Electrocardiography was performed to determine the cardiovascular status of each patient (Figure 2). 

Ophthalmic timolol 0.5% (Sina Darou, Iran) was used to provide the active ingredient of TG. To make timolol gel, the study group twice received distilled and sterilized water and HPMC 4% mixed with preservative and the same volume of the other timolol ingredients. On the other hand, the placebo group took HPMC 2% mixed with the other timolol ingredients except the active ingredient. The appearance and consistency of the two samples were similar (colorless) and could not be differentiated visually.

Microbial and stability control tests were carried out according to USP 42 (2019) standards at the Faculty of Pharmacy of MAZUMS. Finally, the product was prepared as a single dose to prevent microbial contamination during the administration.

The efficacy of 0.25% TG in the transplantation site in the patients undergoing STSG was investigated. The final product was rubbed on the position as a fingertip unit in 2 cm^2^ of the donor site. The first dose was given immediately after surgery (twice daily for the first 48 hours in the hospital, followed by once daily at home) and continued for 14 days.

The usual care of the donor site skin graft was provided for all patients. This included vaseline gauze and, in the case of excessive discharge from the wound, sterile gauze dressing for a limited time.

The primary outcome was re-epithelialization, and the two secondary outcomes were the level of pain on days 1, 2, 3, 4, 7, and 14 based on the Visual Analog Scale (VAS) score (ranging from 0, meaning no pain, to 10, meaning the most severe pain) and the incidence of wound infection. The patients were also evaluated for scar status 3 months after surgery according to the Vancouver Scar Scale (VSS) and the Patient and Observer Scar Assessment Scale (POSAS) (19-21). The VSS is designed based on the physical parameters related to wound healing and maturation, the appearance of wounds, and improved skin function, including several items. Also, the POSAS reflects the supervisors’ observation and patients’ opinions in evaluating scars (19, 22).

The patients’ vital signs were checked upon admission to screen the patients for the side effects. Indeed, if the drug effectively reduces pain, it can affect vital signs (23-25). Pain is associated with increased heart rate, respiratory rate, and blood pressure (26-28). Upon admission and follow-up, the patients were evaluated for possible side effects (such as bradycardia, hypotension, arrhythmia, and dyspnea) (29).

*Statistical Analysis*

The data were analyzed in SPSS 24. Kolmogorov-Smirnov test was used to check the distribution of data, and the descriptive statistics were used to express the baseline and clinical characteristics of patients. The Chi-square test (or Fisher’s exact test) was used to compare the qualitative variables between the two groups. To compare the mean of quantitative variables, we used an independent sample *t*-test or its nonparametric equivalent. Repeated measure ANOVA was also used to compare changes between the two groups over time. The intention to treat analysis was applied, and *P < *0.05 was considered statistically significant.

The sample size was estimated using the results of the study of Mohammadi *et al. *(30). In the present study, the mean and standard deviation of wound healing was 16.13 and 7.40 in the intervention group and 21.52 and 7.94 in the control group. Considering these results, the confidence interval of 95%, power of 80%, and using the two-tailed test and a comparison formula between the means in Stata software, we estimated the sample size at 64 (32 in each group).

## Results

Among the 64 patients included in the study, there were 23 women and 41 men. Participants were randomly divided into the study group (n = 32) and the control group (n = 32).

The details of the study population are given in Table 1. 

There was no significant difference in gender, age, weight, height, hemoglobin, albumin, burn percentage, and total body surface area (TBSA) between the two groups. Similarly, the two groups did not differ significantly with respect to donor size (Table 1).

The Patients were classified according to the cause of their admission, the most common cause of which was hot water burning (23.43%). 

Systolic blood pressure (SBP), diastolic blood pressure (DBP), respiratory rate (RR), and body temperature (T) were different in the two groups on the second day (Table 2). Also, SBP on the fourth day (*P = *0.24) and RR on the third day (*P = *0.006) were different in the two groups. 

However, there was no significant difference between the two groups regarding heart rate (HR) on the second, third, and fourth day.

There was a significant difference in the healing time in the two groups (*P = *0.000) (Table 3).

No infection occurred in either group, and three cases of transplant rejection occurred in the placebo group. Visual Analog Scale (VAS) was statistically significant on days 1, 2, 3, 4, and 7 between the two groups, but it varied only slightly and not significantly on day 14 (*P* = 0.07).

The results of data analysis over time using repeated measure ANOVA showed that DBP (*P = *0.070), T (*P = *0.188), and HR (*P = *0.716) were not significantly different. However, changes over time were statistically significant for SBP (*P = *0.021), RR (*P = *0.007), and VAS (*P < *0.001).

There was no significant difference between the two groups regarding the POSAS scores reported by the patients and the observer in the third month.

On the other hand, the VSS score measured in the third month was statistically significant in the two groups (*P = *0.005). 

## Discussion

The donor site caused by STSG is susceptible to prolonged healing time and increased pain in patients (31, 32). This study showed that administering TG on donor sites positively affects wound healing. Thus, the re-epithelialization time in patients treated with TG was shorter than in those who did not receive it. Also, TG significantly reduced pain on day 14 compared to the baseline. 

Regarding hemoglobin and albumin levels, we did not find a significant difference between the two groups and, consequently, ruled out the effect of nutrition on the duration of wound healing.

**Figure 3:**
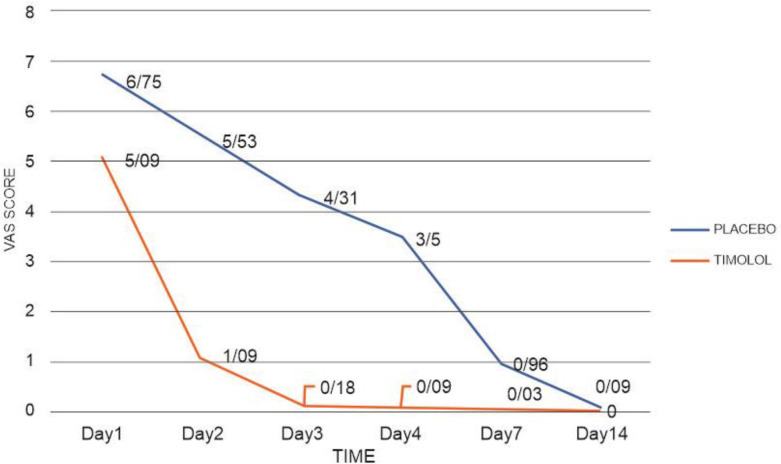
Pain changes regarding Visual Analogue Scale scores (VAS).

VAS is a valid measure for assessing acute pain based on the patient’s conception of pain. It shows a spectrum of pain ranging from “no pain” (scored 0) to “most severe pain” (scored 10) (33). Our data suggest that timolol significantly reduced pain (Figure 3); besides, it significantly affected SBP, DBP, T, and RR on the second day of treatment.

Acute pain amplifies sympathetic activity, which causes blood pressure changes (24). It has been observed that patients with higher diastolic and systolic blood pressure experience greater clinical pain severity (23). Therefore, blood pressure changes may be due to a decrease in pain. In our study, the statistically significant decrease in pain in the TG group could be related to a greater reduction in pain on the second day than on other days. 

Studies show that timolol is absorbed by the skin and may cause side effects or allergies (34, 35).

Nevertheless, we did not observe any complications such as bradycardia.

It seems that body temperature is related to the pain level (36). Surgery sometimes causes difficult conditions, which can lead to fever in the first 48 h after surgery .(25)Perhaps the significant difference in temperature decrease on the second day is due to the reduction of patients’ pain after surgery.

There is some evidence confirming that systemic administration of beta-blockers such as propranolol has beneficial effects on the healing process and recovery time. Moreover, systemic absorption and detectable plasma levels can occur following topical TG use. The lack of transplant rejection in the timolol group could be related to systemic absorption and its effects on the transplant area (30, 34).

Regarding long-term scar assessment, the obtained VSS results showed that timolol improves the appearance of the scar site in the third month (*P = *0.005); this may indicate an increase in keratinocytes migration during the first 3 months after transplantation. On the other hand, the VSS score in the third month was only slightly but not significantly different in the placebo group (*P *= 0.06). This difference could become significant if the sample size increases.

There are many case reports on timolol and chronic wounds, and they suggest the favorable effects of this compound in wound healing. However, the doses used in these studies vary (6, 7, 9, 10 and 13). One such study used 0.1% gel to evaluate the beneficial effects of timolol on acute wounds (4). Several studies have addressed the effect of topical and oral administration of propranolol in acute and chronic cases, and acceptable results have been reported (11, 12 and 30). Meanwhile, several other studies on skin grafts have not used beta-blockers (1, 3). Combining these therapeutic regimens may be a good line for future research.

Our study has some limitations, including the small number of patients admitted to a single center. Due to their distance from the medical center, some patients were followed up by phone and based on their submitted photos.

**Table 1:** Patient demographics and the reason for skin transplant. TBSA: Total Body Surface Area

**Parameter**	**Placebo** **(N = 32)**	**Timolol** **(N = 32)**	***P*** **-value**
Age, year	43 ± 17	46 ± 15	0.49
Sex, males, n (%)	21 (65.6)	20 (62.5)	0.79
Height, cm	169 ± 7	168 ± 9	0.69
Weight, kg	75 ± 14	74.4 ± 14	0.88
Hemoglobin, g/dL	12.4 ± 1.9	12.6 ± 1.9	0.72
TBSA, m^2^	1.86 ± 0.2	1.85 ± 0.2	0.87
Albumin, g/dL	3.6 ± 0.4	3.5 ± 0.4	0.73
Burn, %	10.15 ± 9	10.12 ± 8.7	0.59
**Cause of admission, n (%)**			
Hot water	7 (21.87)	8 (25)	
Gas explosion	8 (25)	2 (6.25)	
Acid	2 (6.25)	2 (6.25)	
Hot solid	5 (15.62)	3 (9.37)	
Gasolin	3 (9.34)	10 (31.25)	
Electrical	1 (3.12)	1 (3.12)	
Reconstructive Surgery	6 (18.75)	6 (18.75)	
Donor Size, cm^2^	236.65 ± 165	230.78 ± 142	0.88

**Table 2:** Vital sign changes during the study period. SBP: Systolic Blood Pressure; DBP: Diastolic Blood Pressure; VAS: Visual Analogue Scale; POSAS: Patient and Observer Scar Assessment Scale

**Vital sign**	**Placebo** **(N = 32)**	**Timolol** **(N = 32)**	***P*** **-value**	***P*** **-value (** **repeated measure ANOVA)**
**SBP (mmHg)**				0.021
Base line	112.1 ± 9.7	111.2 ± 10.5	0.748	
Day 1	112.8 ± 8.5	108.5 ± 10.4	0.097	
Day 2	113.2 ± 9.8	106.7 ± 8.3	0.007	
Day 3	111.5 ± 8.8	107.1 ± 10.1	0.065	
Day 4	112.9 ± 9.4	107.8 ± 9.0	0.024	
**DBP (mmHg)**				0.070
Base line	70.4 ± 7.9	71.2 ± 9.0	0.883	
Day 1	71.8 ± 7.8	68.5 ± 7.0	0.081	
Day 2	72.1 ± 8.3	67.5 ± 7.1	0.021	
Day 3	71.2 ± 7.5	67.5 ± 8.0	0.065	
Day 4	72.1 ± 7.9	69.0 ± 6.8	0.089	
**Body Temperature (°C)**				0.188
Base line	37.1 ± 0.4	37.0 ± 0.4	0..888	
Day 1	36.9 ± 0.3	36.8 ± 0.3	0.328	
Day 2	36.9 ± 0.4	36.6 ± 0.3	0.002	
Day 3	36.8 ± 0.3	36.8 ± 0.4	0.389	
Day 4	36.7 ± 0.3	36.7 ± 0.3	0.388	
**Respiratory Rate (n/min)**				0.007
Base line	16.5 ± 2.9	17.7 ± 2.5	0.066	
Day 1	17.9 ± 1.5	18.3 ± 0.8	0.091	
Day 2	17.6 ± 1.2	18.4 ± 0.9	0.008	
Day 3	17.3 ± 1.6	18.3 ± 0.6	0.006	
Day 4	17.6 ± 1.2	18.2 ± 0.8	0.051	
**Heart Rate (n/min)**				0.716
Base line	79.3 ± 7.1	81.8 ± 4.5	0.050	
Day 1	80 ± 4.0	78.4 ± 3.0	0.115	
Day 2	79.9 ± 3.8	78.6 ± 2.2	0.089	
Day 3	79.3 ± 3.5	79 ± 2.0	0.884	
Day 4	79 ± 3.6	78.4 ± 2.0	0.514	

**Table 3:** Clinical efficacy and safety variables. VAS: Visual Analogue Scale; VSS: Vancouver Scar Score; POSAS: The Patient and Observer Scar Assessment Scale

**Parameter**	**Placebo** **(N = 32)**	**Timolol** **(N = 32)**	***P*** **-value**	***P*** **-value (** **repeated measure ANOVA)**
**VAS**				<0.001
Day 1	6.75 ± 2.1	5.09 ± 2.1	0.004	
Day 2	5.53 ± 1.8	1.09 ± 1.4	<0.001	
Day 3	4.31 ± 1.7	0.18 ± 0.5	<0.001	
Day 4	3.5 ± 1.8	0.09 ± 0.2	<0.001	
Day 7	0.96 ± 1.1	0.03 ± 0.1	<0.001	
Day 14	0.09 ± 0.2	0	0.078	
Healing Time, Day	14.5 ± 3.2	11.5 ± 2.3	<0.001	-
Graft Rejection, N	3	0	-	-
Infection, N	0	0	-	-
POSAS, Patient	4.78 ± 1.5	4.18 ± 1.2	0.12	-
POSAS, Observer	3.68 ± 1.1	3.15 ± 1.1	0.06	-
VSS, Month 3	4.75 ± 1.9	3.34 ± 1.8	0.005	-

## Conclusion

The favorable effect of topical TG on wound healing and pain reduction with an acceptable safety profile and low cost can make it a potential therapeutic agent in patients with a skin graft.
